# Can Butyrate offer a novel approach to mitigate the pathology of AD?

**DOI:** 10.1002/alz.088636

**Published:** 2025-01-03

**Authors:** Warnakulasuriya Mary Ann Dipika Binosha Fernando, Ian J Martins, Uththara Senerath, Prashant Bharadwaj, Ralph N Martins

**Affiliations:** ^1^ Australian Alzheimer’s Research Foundation, Perth, Western Australia Australia; ^2^ Edith Cowan University, Perth Australia; ^3^ Centre of Excellence for Alzheimer’s Disease Research and Care, School of Medical and Health Sciences, Edith Cowan University, Joondalup, Western Australia Australia; ^4^ Edith Cowan University, Perth, Western Australia Australia; ^5^ Co‐operative research Centre (CRC) for Mental Health, Carlton South, VIC Australia; ^6^ McCusker Foundation for Alzheimer’s Disease Research and Care, Joondalup Australia; ^7^ Centre of Excellence for Alzheimer’s Disease Research and Care, School of Medical and Health Sciences, Edith Cowan University, Perth Australia; ^8^ ECU, Perth, Western Australia Australia

## Abstract

**Background:**

Our research group is currently exploring the potential of Butyric acid (NaB), a Short Chain Fatty Acid (SCFA), as a novel therapeutic agent for Alzheimer’s disease (AD).

**Methods:**

In our investigation using the 5xFAD mouse model of AD, we observed that NaB had significant effects on Aβ levels, as well as on associative learning and cognitive functioning. Notably, we recorded a 40% reduction in brain Aβ and a 25% increase in fear response during both cued and contextual testing. Our studies employing M17 cell models demonstrated that butyrate provides a degree of protection against Aβ‐induced toxicity. Additionally, nanoindentation studies confirmed that butyrate induces changes in cell physiology. Building on these findings, we explored the impact of butyrate on astrocytes and microglial cells concerning amyloid beta toxicity.

**Results:**

Current studies with stem cells have exhibited promising outcomes, indicating that butyrate significantly alleviates amyloid beta.

**Conclusions:**

This investigation represents the initial confirmation of butyrate as an amyloid beta inhibitor across various Alzheimer’s disease (AD) models. The subsequent steps involve determining the levels of butyrate at different stages of AD by analyzing fecal samples and conducting tests to assess its therapeutic efficacy.

